# Ultralow charge-transfer resistance with ultralow Pt loading for hydrogen evolution and oxidation using Ru*@*Pt core-shell nanocatalysts

**DOI:** 10.1038/srep12220

**Published:** 2015-07-20

**Authors:** Jia X. Wang, Yu Zhang, Christopher B. Capuano, Katherine E. Ayers

**Affiliations:** 1 Department of Chemistry, Brookhaven National Laboratory, Upton, NY 11973, USA; 2 Proton OnSite, 10 Technology Drive, Wallingford, CT 06492 USA

## Abstract

We evaluated the activities of well-defined Ru@Pt core-shell nanocatalysts for hydrogen evolution and oxidation reactions (HER-HOR) using hanging strips of gas diffusion electrode (GDE) in solution cells. With gas transport limitation alleviated by micro-porous channels in the GDEs, the charge transfer resistances (CTRs) at the hydrogen reversible potential were conveniently determined from linear fit of ohmic-loss-corrected polarization curves. In 1 M HClO_4_ at 23 °C, a CTR as low as 0.04 Ω cm^−2^ was obtained with only 20 μg cm^−2^ Pt and 11 μg cm^−2^ Ru using the carbon-supported Ru@Pt with 1:1 Ru:Pt atomic ratio. Derived from temperature-dependent CTRs, the activation barrier of the Ru@Pt catalyst for the HER-HOR in acids is 0.2 eV or 19 kJ mol^−1^. Using the Ru@Pt catalyst with total metal loadings <50 μg cm^−2^ for the HER in proton-exchange-membrane water electrolyzers, we recorded uncompromised activity and durability compared to the baseline established with 3 mg cm^−2^ Pt black.

The hydrogen evolution and oxidation reactions (HER-HOR) are a pair of important reactions for carbon-free energy conversion - producing hydrogen in water electrolyzers and generating electrical power in hydrogen fuel cells. While platinum (Pt) nanoparticles are highly active catalysts for both HER and HOR, the scarcity and high cost of Pt impede large-scale commercialization of these clean energy technologies^1^,^2^,^3^,^4^. For proton exchange membrane (PEM) fuel cells, uncompromised HOR performance with Pt loading as low as 50 μg cm^−2^ was achieved in recent years^4^,^5^. However, Pt loading <200 μg cm^−2^ remained elusive for the HER, without incurring a performance penalty in PEM water electrolyzers^6^,^7^,^8^.

Our approach controls core-shell structure at the atomic level to maximize the Pt specific surface area and to improve catalytic performance through core-metal-induced effects. An economically viable method was recently developed to synthesize single crystalline Ru@Pt core-shell nanoparticles that exhibited an atomically sharp core-shell interface^9^. In PEM fuel cells, we found that the ordered Pt bilayer shells prevented the dissolution of the less noble Ru core in acid at the potentials up to 0.95 V, and exhibited enhanced tolerance to carbon monoxide^9^,^10^. Here, we report that uncompromised HER performance in PEM water electrolyzers with low Pt loading, <50 μg cm^−2^, was achieved using the Ru@Pt (atomic ratio 1:1, bilayer thick Pt shell) nanocatalysts. The optimal Pt shell thickness and the minimal Pt loading level for top performance were determined by tests in acid solutions using hanging strips of gas diffusion electrodes (GDEs).

The rotating disk electrode (RDE) method is commonly used for studying and comparing electrocatalysts under standardized conditions; the main drawback is the limitation of the current density, which is on the scale of mA cm^−2^. The GDE method described in this report removes such limitation, which is particularly important for studying fast reactions, such as the HER-HOR on active catalysts in acids. Traditionally, the RDE method was used for determining the HOR activities on smooth Pt crystal surfaces^11^,^12^ and on thin nanocatalyst films^13^. However, the RDE’s limiting currents on the scale of mA cm^−2^ are insufficient for unambiguously determining the high HER-HOR activity on Pt in acids^14^, as was later found by microelectrode measurements^15^ and hydrogen pump experiments in PEM fuel cells^5^. Other methods, such as using a porous electrode floating on the electrolyte solution, also were found effective in enhancing gas transport, and thus, were used in studying the intrinsic kinetics of the HOR and the oxygen reduction reaction on Pt nanocatalysts^16^.

Less noticed was that the HER activity also is profoundly affected by gas transport, even though H_2_ is the product, not the reactant as in the HOR. We illustrate this fact here by comparing the polarization curves measured on a RDE and a hanging strip of GDE. The latter affords us a feasible method to alleviate gas transport limits in solution electrochemical cells, and to quantify the HER-HOR activity by measured charge transfer resistance (CTR) at the reversible potential of 0 V. We used the same gas diffusion layers as in PEM water electrolyzers and fuel cells, and thus, the results of these low-cost, time-saving solution tests guided us in optimizing the atomic ratio of Ru:Pt and determining the level of loading required for top performance in real devices.

## Results and Discussion

**Figure 1:**
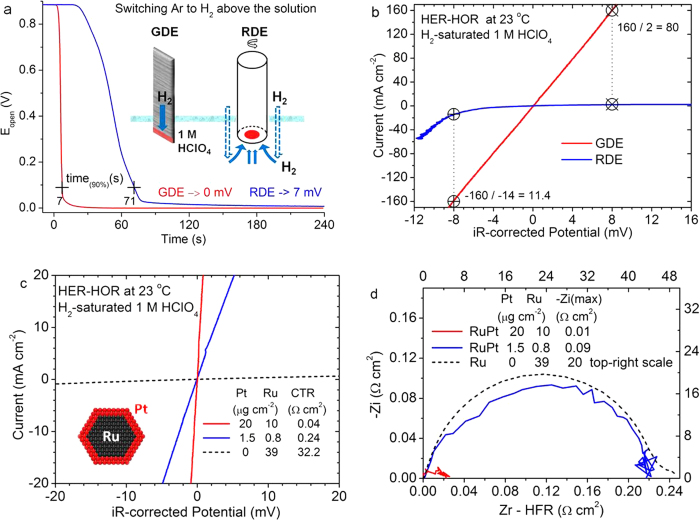
HER-HOR activity measured using hanging-strip GDEs in 1 M HClO_4_ at 23 °C. (**a**) Open circuit potentials on a GDE and an RDE (2500 rpm) as a function of time after the gas inlet above the 1 M HClO_4_ solution was switched from Ar to H_2_. The arrows in the insert indicate different paths of hydrogen gas to the catalysts on a GDE and a RDE. (**b**) HER-HOR polarization curves on a GDE and an RDE (2500 rpm), both having an area of 0.2 cm^2^ with the same amount of Ru@Pt catalysts (20 μg cm^−2^ Pt, 11 μg cm^−2^ Ru). (**c**) HER-HOR polarization curves and (**d**) impedance spectra of three typical GDE samples (Electrode area: w × h = 1.4 × 0.7 = 1 cm^2^).

As illustrated in the insert of Fig. 1a, we coated catalysts on one side and at one end of a rectangular gas diffusion layer, and held such a strip vertically, with its catalyzed part immersed in an electrolyte solution. A Pt flag placed face-to-face with the GDE strip acted as the counter electrode. To easily make a comparison with the RDE method, we made a GDE sample with the same 0.2 cm^2^ electrode area as the RDE. A remarkable enhancement in the rate of gas diffusion was directly evident by following the changes in the measured open-circuit potentials after switching the gas above the solution from Ar to H_2_. The curves in Fig. 1a show that a 90% decrease of the open-circuit potential from 0.88 to 0.088 V (zero is defined with a hydrogen-saturated solution) takes 7 seconds on a GDE, ten times quicker than on an RDE, illustrating the effectiveness in enhancing gas transport via microporous channels inside the GDE. These gas channels not only speed up gas-saturation of the solution, most importantly, they supply hydrogen gas directly to the catalysts on the GDE during the HOR. In contrast, hydrogen gas firstly dissolves into solution and then diffuses through liquid to reach the catalysts on the RDE, which are slow, and thus, limit the HOR currents (See the path indicated by the arrows in the insert of Fig. 1a).

The huge impact of enhanced gas transport on the HER-HOR kinetics is shown by the ohmic-loss-corrected polarization curves for the GDE and RDE in Fig. 1b. The high frequency resistance (HFR) determined by impedance measurements at 0 V was used for correcting the ohmic loss that is also termed as iR drop (current × resistance) in voltage. While it is well known that starvation of reactant H_2_ causes leveling off of the HOR current at a few mA cm^−2^ on the RDE, the comparison in Fig. 1b further reveals that the HER current is also severely reduced on the RDE due to slow removal of the product, H_2_. As an example, the HER current at −8 mV on the GDE is 11-fold of that on the RDE; although this is less than the 80-fold for the HOR current at 8 mV, an-order-of-magnitude difference shows that the HER activity of a highly active catalyst may be significantly underestimated using the RDE method.

The linear, symmetric polarization on the GDEs near 0 V affords us a simple way to quantify the HER-HOR activity. Figure 1c shows three iR-corrected polarization curves for the carbon-supported Ru@Pt and Ru nanocatalysts, in which the CTR values were determined by linear fits of the iR-corrected curves or equally by subtracting the HFRs from the total resistances obtained from linear fits of measured polarization curves. A lower CTR represents a higher activity, which can be correlated with the exchange current using *j*_*0*_ (A cm^−2^) = 0.0257 (V)/CTR (Ω cm^2^), where 0.0257 is the value of the constant RT/F at 25 °C^17^,^18^. In comparison with a CTR of 32.2 Ω cm^2^ for the Ru nanoparticles, the CTRs were reduced to 0.24 and 0.04 Ω cm^2^ using the Ru@Pt catalysts (Ru:Pt atomic ratio 1:1) with only 1.5 and 20 μg cm^−2^ Pt, respectively. The activity difference of more than two orders of magnitude is also reflected in the corresponding impedance plot, where the data for Ru nanoparticles was plotted using the top-right scale that differs by orders of magnitude from the bottom-left axes in Fig. 1d. Besides demonstrating that a wide range of activity can be directly measured on GDEs in a solution electrochemical cell, including the data for a Ru/C sample here clarifies that the contribution to the HER-HOR activity from exposed Ru surface is negligible for Pt sub-monolayer catalysts.

**Figure 2:**
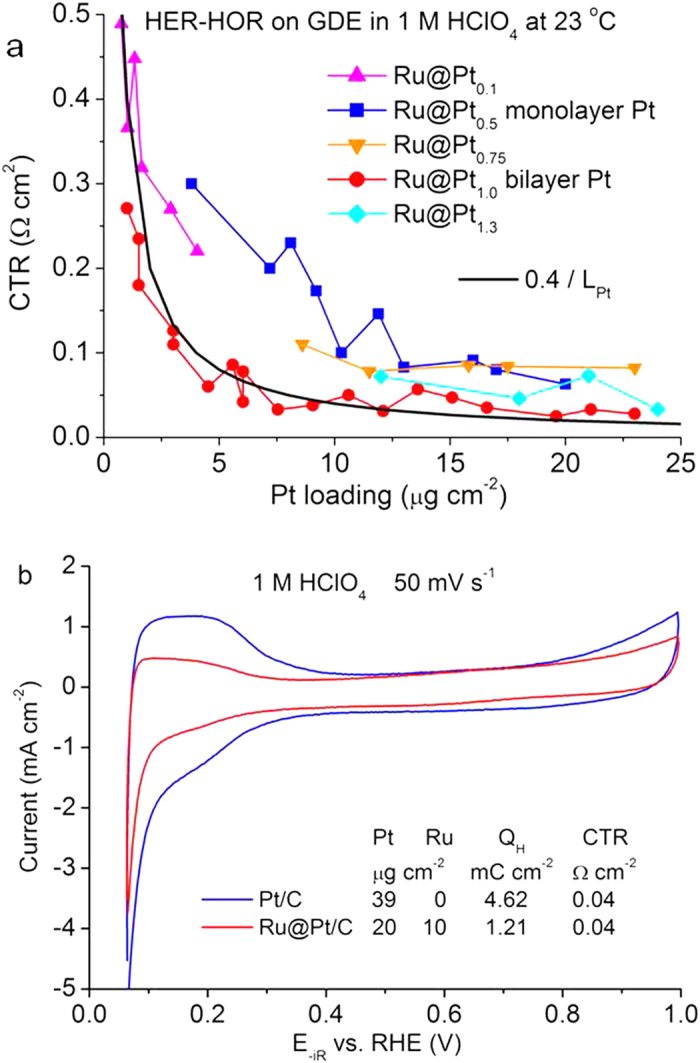
Pt-loading-dependent HER-HOR CTR and cyclic voltammetry curves measured using hanging-strip GDEs. (**a**) CTRs measured in hydrogen saturated 1 M HClO_4_ at 23 °C for five Ru@Pt catalysts with the Pt:Ru atomic ratio ranging from 0.1 to 1.3 as a function of Pt loading. The black line is calculated using 0.4 (Ω cm^2^) divided by Pt loading L_Pt_ (μg cm^−2^). (**b**) iR-corrected voltammetry curves on the GDE strips for a Pt and a bilayer Ru@Pt_1.0_ catalysts.

To determine the optimal Pt:Ru atomic ratio in the Ru@Pt catalysts and the minimal Pt loading that is required for top performance, we measured the Pt-loading-dependent CTRs for five Ru@Pt catalysts with the Pt:Ru atomic ratios ranging from 0.1 to 1.3. In our previous studies, monolayer and bilayer Pt shells, respectively, were found for the Ru@Pt catalysts synthesized using 0.5 and 1.0 Pt:Ru atomic ratio^9^. With Pt loadings up to 25 μg cm^−2^, the best performance often was attained with the bilayer Ru@Pt_1.0_ catalyst (red dots in Fig. 2a). The trend is illustrated by a calculated curve using CTR (Ω cm^2^) = 0.4/Pt loading (μg cm^−2^), that is, ≤0.04 Ω cm^2^ can be obtained with ≥10 μg cm^−2^ Pt. Due to the uncertainty level in determining the HFR (see the noise in Fig. 1d for highly active samples), we consider 0.04 Ω cm^2^ as the minimal value that can be unambiguously determined.

Using commercial Pt catalysts, we obtained similar CTRs with the Pt loadings twice as those when the Ru@Pt_1.0_ catalyst was used, indicating a 50% reduction in Pt loading via the bilayer-thick core-shell structure. We attribute this doubling in Pt mass activity to the approximate doubling of the Pt specific surface area for the bilayer Ru@Pt_1.0_ catalyst (1.0 cm^2^ μg^−1^) compared to that for the Pt catalyst (0.56 cm^2^ μg^−1^). These values were obtained by calculation using the average particle sizes and measured CO stripping charges^10^. It is worth noting that hydrogen adsorption/desorption charge measured for the bilayer Ru@Pt_1.0_ nanocatalyst is significantly less than that for the Pt nanocatalyst while their calculated surface areas are similar (Fig. 2b). This is caused by the influence of the Ru core on the Pt surface as it is known that adsorptions of H and OH are weakened on Ru-supported Pt films with ≤3 monolayer thickness^19^,^20^,^21^,^22^.

A question may rise on why a significant weakening of H adsorption by the influence of the Ru core had little impact on the specific activity of the Pt shell for the HER-HOR in acids. Two opposite effects on the adsorption energy of the reaction intermediate is likely the answer. Infrared spectroscopic studies identified atop-site-adsorbed H atom, H_atop_, as the active intermediate of the HER-HOR on Pt^23^, whose binding is weakened by the lateral repulsion with H adsorbed at bridge or hollow sites^24^. The latter also is called underpotentially deposited hydrogen, H_upd_, as it originates from proton reduction from solution. While the direct effect of a Ru core weakens H adsorption at all sites, the lowered H_upd_ coverage by about fourfold (Q_H_ ratio of 3.8 in Fig. 2b) reduces lateral repulsion^24^, which indirectly strengthens the adsorption of the active HER-HOR intermediate, H_atop_. Thus, the net effect of a Ru core on the specific activity for the HER-HOR in acid becomes negligibly small. This explanation is supported by our determined apparent activation barrier for the Ru@Pt_1.0_ catalysts being similar to that reported for Pt catalysts as described below.

**Figure 3:**
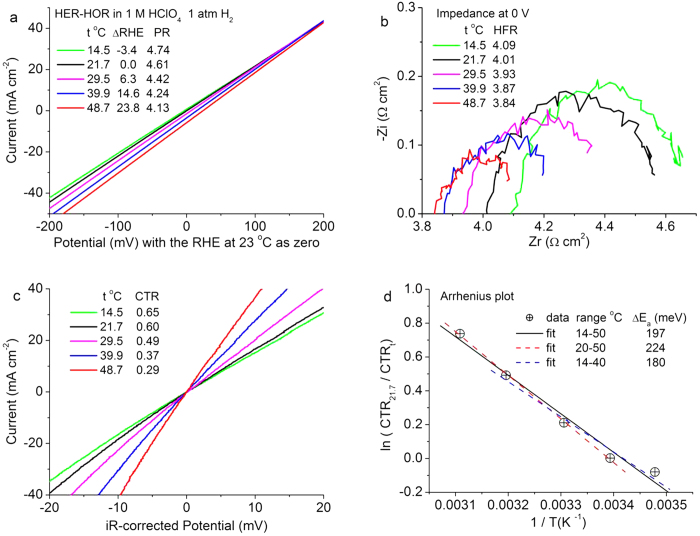
HER-HOR activation barrier determined from temperature-dependent CTR for the Ru@Pt_1.0_ catalyst on a GDE in hydrogen-saturated 1 M HClO_4_ solution. (**a**) Measured polarization curves showing the change in the reversible hydrogen potential (RHE) and polarization resistance (PR), (**b**) impedance spectra with temperature dependent HFRs, (**c**) iR-corrected polarization curves with temperature dependent CTRs, (**d**) Arrhenius plot, activation barrier, ΔE_a_ = 0.20 ± 0.02 eV = 19 kJ mol^-1^.

Temperature-dependent CTRs were measured using a Ru@Pt_1.0_ catalyst supported on carbon nanotubes because a network of nanotubes is less prone to fall off than carbon powders during the measurements. Figure 3a plots measured polarization curves at several temperatures. From the potential shifts at zero current, we found an increase of the reversible hydrogen potential, *E*_*0*_, at a rate of 0.8 mV per °C (by linearly fitting the data in Fig. 3a). The polarization resistance (PR) measured from the slope also change vary with temperature, which are the sum of CTR and HFR. The HFRs determined from the intercept with the Zr axis in Fig. 3b show a decrease with temperature of −7.2 mΩ cm^2^ per °C (by linearly fitting the data in Fig. 3b). The iR-corrected polarization curves in Fig. 3c illustrate the change in slope corresponding to the decrease in the CTR with increasing temperature. The activity for the HER-HOR represented by 1/CTR is normalized to the value at 21.7 °C in the Arrhenius plot (Fig. 3d). The linear fit yields an apparent activation barrier of 0.20 ± 0.02 eV, or 19 ± 2 kJ mol^−1^, which is close to 20.6 kJ mol^−1^ for polycrystalline Pt^25^ and 18 kJ mol^−1^ for the Pt(111) surface^12^. This activation barrier indicates that the CTRs can be lowered by a factor of 1.9 and 3.5, respectively, at 50 °C and 80 °C vs. those measured at 23 °C.

**Figure 4:**
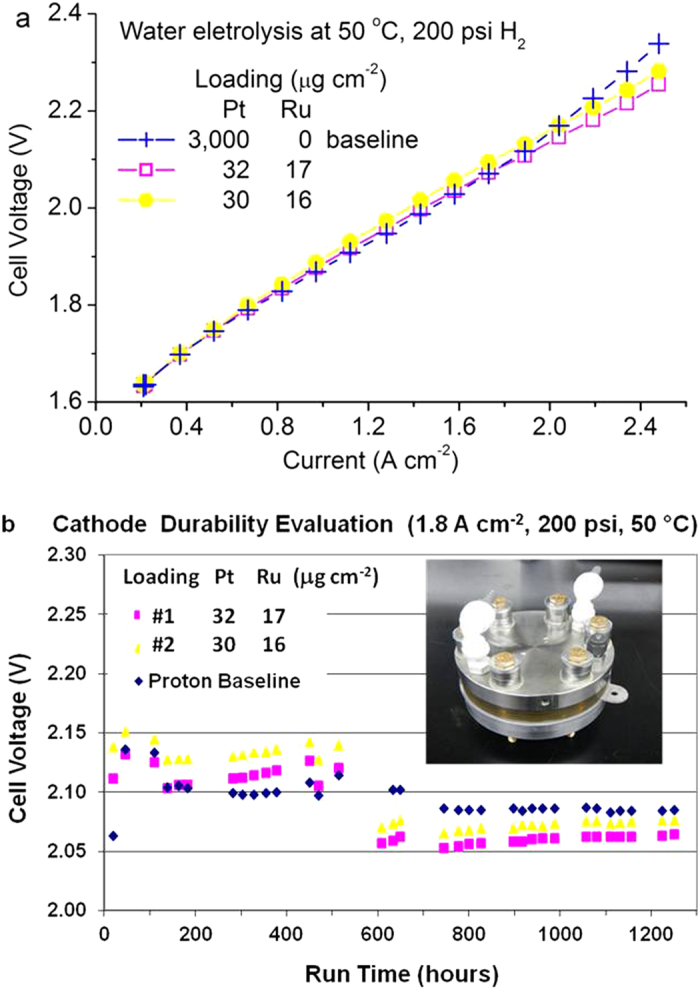
HER performance tests in PEM water electrolyzers. (**a**) Polarization curves and (**b**) voltage stabilities measured at 1.8 A cm^−2^ for two cathode samples made of the Ru@Pt_1.0_ core-shell catalysts and one made of Pt black catalysts as the baseline using a three-cell stack (inset of (**b**)).

We tested the HER performance of the bilayer Ru@Pt nanocatalysts in PEM water electrolyzers at Proton OnSite. Among sixteen samples tested, the GDEs that performed similarly as the baseline had solution-tested CTR ≤0.08 Ω cm^2^. Figure 4a,b show the results of a three-cell stack test. Two of the three cathodes were made with the Ru@Pt_1.0_/C catalysts, and one with Pt black as the baseline. The polarization curves and time-dependent cell voltages were measured for each of the three cells. A lower cell voltage at a given current represents a higher HER activity. The polarization curves in Fig. 4a show that the two Ru@Pt/C samples exhibited equal or slightly better (at >2 A cm^−2^) activities to the baseline. During the durability test carried out at 1.8 A cm^−2^, an unplanned system shutdown occurred at about 600 hours; after restarting the system, the cell voltages lowered by 0.02 to 0.06 V for the three cells without an apparent reason. Despite this incident, all three curves are rather flat over 1200 hours (Fig. 4b), showing no sign of degradation. These results demonstrated the total metal loading (65% Pt and 35% Ru) can be lowered to 50 μg cm^−2^ in the cathode of PEM water electrolyzers without penalty in performance and durability.

In conclusion, the bilayer Ru@Pt core-shell catalyst is validated as a practical, high performance, low-cost HER-HOR nanocatalyst, which can help in cost reduction, and thus, in enabling large-scale commercialization of PEM water electrolyzers and fuel cells. A benchmark activity of ≤0.04 Ω cm^2^ CTR for the HER-HOR at 0 V in 1 M HClO_4_ at 23 °C with Pt loading ≤20 μg cm^−2^ is established using the GDE testing method in solution electrochemical cells. We attributed the ultralow CTR with ultralow Pt loading to the high Pt surface area per Pt mass of the well-defined Ru@Pt core-shell catalysts and optimized GDE fabrication. More mechanistic discussion will be published in future that compares the HER-HOR polarization behavior on GDE strips in acid and alkaline solutions.

## Methods

### Preparation of catalyst inks

Experimental details for synthesizing single crystalline Ru@Pt core-shell nanocatalysts on carbon supports, Ru@Pt/C, have been described elsewhere^9^. In both processes of making carbon support Ru core nanoparticles and subsequent coating Pt shells on the Ru cores, the weight of the final catalysts are consistent to the sum of the weight of metal precursors and carbon support (Ketjenblack EC-600JD). Thus, the weight percentages for Pt and Ru in carbon supported catalysts were calculated from the amounts of metal precursors used with a given amount of carbon in synthesis.

Catalyst inks were prepared by dispersing the carbon-supported catalysts in mixed solvents of deionized water, iso-propanol, ethanol and Nafion^®^ (perfluorinated resin, equivalent weight 1000, Aldrich). Nafion is both a binder and an ionomer. We obtained similar results in solution tests with Nafion/carbon weight ration ranging from 0.5 to 1.0, and used the ratio of 1.0 for the results shown in this report. The volumes of water, iso-propanol, and ethanol, respectively, were 40, 80, and 40 μL for 1 mg carbon. Since pouring alcohol on dry Pt-containing nanocatalysts may cause sparking, we added water first and ethanol last. The mixture was sonicated in an ice bath for 5–10 min and mixed by shaking for at least 1 hour using Mixer Mill MM 400 (Retsch). The inks were sonicated again before each use.

For determining metal loadings based on ink volume, we calculated Pt and Ru concentrations from the amount of catalyst and total volume of solvents. We also calculated the Pt (Ru) weight percentage (wt%) in the catalyst ink excluding solvents, which is the dry mass of catalyst and Nafion. With an equal weight of Nafion as the carbon, we divided the Pt (Ru) wt% in the catalyst by (1+ carbon wt% ). For example, a Ru@Pt_1.0_/C catalyst was made with 200 μmol Ru and 200 μmol Pt per 100 mg carbon, yielding a catalyst containing 24.5 wt% Pt, 12.7 wt% Ru, and 62.8 wt% C. The dry mass of the ink then contains 15 wt% Pt and 7.8 wt% Ru, balance by equal weight of Nafion and carbon. These metal weight percentages for Ru@Pt/C+Nafion were used for calculating the metal loadings based on the weight increase of the GDL after catalyst inks completely dried.

### Preparation of gas diffusion electrodes

The gas diffusion layer (GDL) used in this work was Sigracet GDL 25 BC from Ion Power. We made GDE strips with 1 × 0.2 = 0.2 cm^2^ or 1.4 × 0.7 = 1 cm^2^ area of catalyst coating on 1 or 1.4 cm wide and about 4 cm long GDL strips. Most samples were made by brushing catalyst inks on one side of the strips at one of their ends. To determine the metal loadings, we weighed the strips before coating catalysts and after the solvents had evaporated completely in air at room temperature. The increase in weight corresponds to the total weight of catalyst and Nafion, from which the Pt and Ru loadings were calculated using the metal weight percentages in the catalyst and Nafion mixture. Alternatively, catalyst inks can be drop casted using a pipette with accurate volume and the metal loadings are then calculated by the volume of ink and with the metal concentrations of the ink.

### Solution tests

We held a GDE strip vertically with its catalyzed end completely but not too deeply immersed in the solution, and positioned such that the catalyzed side faces a Pt-flag counter electrode at a distance about 1.5 cm. High concentration solution, 1 M HClO_4_, was used to keep HFR low. Polarization curves for the HER-HOR were obtained in hydrogen-saturated electrolytes by averaging the curves in positive and negative potential sweeps at 20 mV s^−1^. They represent steady-state polarization curves, as we verified by time-dependent measurements after a potential step. Electrochemical impedance spectra were acquired at 0 V with a peak-to-peak perturbation of 20 mV at an ac frequency ranging from 10 kHz to 0.1 Hz. After performing HER-HOR measurements on the GDEs, the electrochemical cells were purged with Ar or N_2_ before taking the GDE strip out of the solution to avoid possible burning when the hydrogen inside the porous GDE is mixed with atmospheric oxygen.

### Membrane electrode assembly tests

We carried out the tests in water electrolyzers using a customized test station fabricated at Proton OnSite specifically for characterizing the cell materials. The test station had an integrated water purification module that maintained on-board conductivity near 18 MΩ cm. A Teflon coated submersible heater controlled the temperature, and all operational tests were conducted at 50 °C. The hardware of a commercial test station for fuel cells was modified for electrolysis tests by replacing the carbon-based flow fields on the cell’s anode side with the ones made of titanium that were designed and fabricated at Proton OnSite. This test-cell hardware was validated against the designs of Proton OnSite’s commercial stacks, so to predict full-scale operational performance. We used a current-control Sorensen power supply to power the cell stack, with over-current protection set at 2.0 A cm^−2^. The current was adjusted through the scan region and allowed to stabilize for 5 minutes before measuring cell potentials.

## Additional Information

**How to cite this article**: Wang, J. X. *et al.* Ultralow charge-transfer resistance with ultralow Pt loading for hydrogen evolution and oxidation using Ru@Pt core-shell nanocatalysts. *Sci. Rep.* **5**, 12220; doi: 10.1038/srep12220 (2015).
